# Synthesis and Characterization of Aminoamidine-Based Polyacrylonitrile Fibers for Lipase Immobilization with Effective Reusability and Storage Stability

**DOI:** 10.3390/ijms24031970

**Published:** 2023-01-19

**Authors:** Yasser M. Al Angari, Yaaser Q. Almulaiky, Maha M. Alotaibi, Mahmoud A. Hussein, Reda M. El-Shishtawy

**Affiliations:** 1 Chemistry Department, Faculty of Science, King Abdulaziz University, Jeddah 21589, Saudi Arabia; 2 Department of Chemistry, College of Science and Arts at Khulis, University of Jeddah, Jeddah 21921, Saudi Arabia

**Keywords:** polyacrylonitrile, hexamethylene diamine, glutaraldehyde, lipase, immobilization

## Abstract

Lipases are extensively utilized industrial biocatalysts that play an important role in various industrial and biotechnological applications. Herein, polyacrylonitrile (PAN) was treated with hexamethylene diamine (HMDA) and activated by glutaraldehyde, then utilized as a carrier support for *Candida rugosa* lipase. In this regard, the morphological structure of modified PAN before and after the immobilization process was evaluated using FTIR and SEM analyses. The immobilized lipase exhibited the highest activity at pH 8.0, with an immobilization yield of 81% and an activity of 91%. The optimal pH and temperature for free lipase were 7.5 and 40 °C, while the immobilized lipase exhibited its optimal activity at a pH of 8.0 and a temperature of 50 °C. After recycling 10 times, the immobilized lipase maintained 76% of its activity and, after 15 reuses, it preserved 61% of its activity. The lipase stability was significantly improved after immobilization, as it maintained 76% of its initial activity after 60 days of storage. The calculated Km values were 4.07 and 6.16 mM for free and immobilized lipase, and the Vmax values were 74 and 77 μmol/mL/min, respectively. These results demonstrated that synthetically modified PAN is appropriate for immobilizing enzymes and has the potential for commercial applications.

## 1. Introduction

Lipases [EC 3.1.1.3] belong to the α/β-hydrolase superfamily’s serine hydrolase class and have the distinctive amino acid triad Ser-Asp (Glu)-His in the active site. They are the most studied enzymes with bioprocess applications [1,2]. Lipases can catalyze a wide range of reactions, including transesterification, interesterification, aminolysis, and acidolysis, while being most frequently utilized for the hydrolysis and esterification of oils and fats [3,4,5]. Lipases have been successfully used in biotechnology and the industry to catalyze various reactions, in relation to food, biomedicine, cosmetics, biosensors, biodegradation, detergent, paper, leather, fuel, structured lipids, flavor esters, and biodiesel production [6,7,8,9,10,11,12,13,14,15,16]. Due to a significant characteristic that enables this enzyme to operate effectively in the presence of organic solvents, lipase’s commercial relevance is related to its ability to promote chemical reactions in both hydrophobic and hydrophilic media [17,18]. Lipases are beneficial selective enzymes that can function as highly flexible catalysts in industrial biotechnology. However, they have some limitations in their free form, like other enzymes. Product separation is hampered by the limited operating stability, high costs, and difficult recovery or re-utilization of free lipases [19,20,21].

The immobilization of enzymes on stable supports offers a remedy for these restrictions. Recently, enzyme immobilization techniques have increased significantly, primarily to improve biocatalysts’ stability and simplify the recovery/reuse stages [22,23,24]. The ability to immobilize enzymes on various solid materials has been studied [25,26,27,28,29]. Although immobilizing enzymes on organic materials allows them to maintain a high activity, these materials lack the necessary chemical and thermal stability for commercial use [30]. Organic–organic hybrid materials can be employed to overcome these challenges. Lipase supports may be made from a variety of polymers, both synthetic and biological in nature. In addition, these polymers may be joined to combine their useful characteristics and produce materials with enhanced attributes that are suitable as lipase supports and for technological applications. Organic–organic hybrids can be created by incorporating (i) two synthetic materials, such as nylon with polyethyl acrylate, polyacrylonitrile with hydrazine hydrochloride, acrylic fibers with hydroxylamine hydrochloride [31,32,33]; (ii) a synthetic polymer with a biopolymer, such as polyester fabric with chitosan and chitosan–polyacrylic acid [34,35]; and (iii) two biopolymers, such as alginate with chitosan [36].

The production of a hybrid that can be used as a lipase support and has the desired properties, such as reusability, stability, and retention of high catalytic efficiency, can be achieved by combining a polymeric material offering thermal resistance, pH stability, and mechanical strength with biodegradable polymers offering biocompatibility and favorable affinity to enzymes [37]. An efficient support for enzyme immobilization based on polyacrylonitrile was produced in our laboratory [38]. A carrier should typically have poor solubility to avoid product contamination and a high surface area to facilitate immobilizing sizable amounts of the enzyme [39]. Different textile fibers have been considered suitable support materials for immobilizing biocatalysts. Due to their strength, affordability, surface area, porosity, pore size, availability in various forms, and simplicity of the functionalization processes, textiles are the material of choice for many applications [40]. In previous work, amidoximated acrylic microfibers were utilized for α-amylase immobilization [33], nonwoven polyester fabric coated with chitosan was used for horseradish peroxidase immobilization [34], and chitosan–polyacrylic acid was utilized for laccase immobilization [35]. In the current work, polyacrylonitrile (PAN) was modified with hexamethylenediamine (HMDA) and activated with crosslinked glutaraldehyde. The modified PAN was utilized as an effective support for the lipase enzyme. The properties of the immobilized enzyme, such as its activity, the effect of temperature, the optimal pH, and the kinetic parameters, were investigated.

## 2. Results and Discussion

### 2.1. Characterization of Modified Polyacrylonitrile

**Figure 1:**
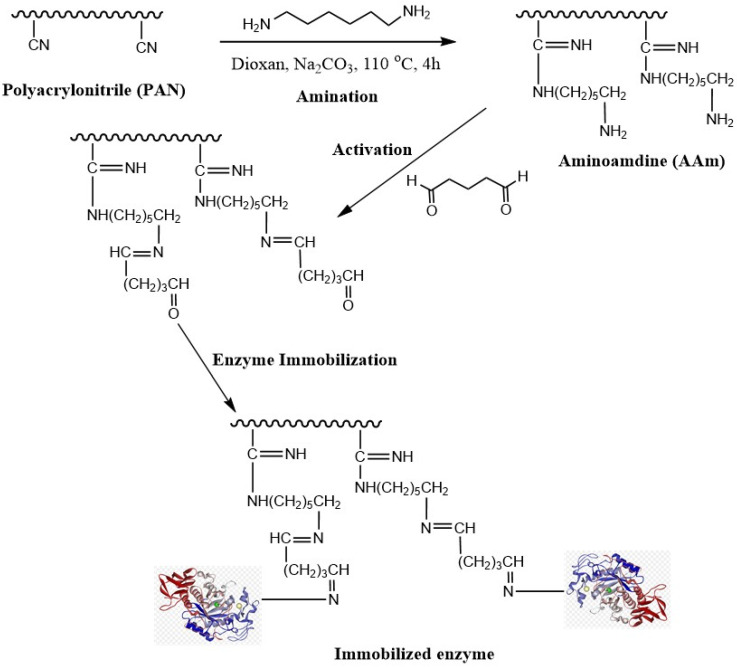
Schematic representation of the modified PAN and immobilized lipase.

Polyacrylonitrile (PAN) has attracted much interest because of its exceptional properties, such as thermal stability and solvent tolerance. For the creation of functional supports, polyacrylonitrile (PAN) was used as the basis matrix. The immobilization strategy involved the amination of the membrane surface containing the nitrile group, the activation with glutaraldehyde, and finally, the immobilization of the lipase enzyme. As shown in Figure 1, the nitrile groups of PAN underwent a reductive conversion with HMDA to aminoamidine (AAm), and the weight increased by 18.6%, confirming the efficacy of the nitrile reduction. Then, different concentrations of glutaraldehyde (1–2.5%) were utilized as a covalent crosslinker for the lipase enzyme. The immobilization yield and recovered activity decreased as the glutaraldehyde concentration increased from 1 to 2.5% (*v*/*v*). The excess glutaraldehyde may have resulted in the denaturation of the enzyme, which may be the reason for this decrease in enzymatic activity [41]. Various-pH buffers were used during the immobilization procedure (Table 1). It should be noted that the immobilized enzyme in this investigation showed its highest activity at pH 8.0. With 1% (*v*/*v*) glutaraldehyde, the immobilization yield (IY%) was 81%, while the activity yield (AY%) was 91%. With the glutaraldehyde concentration increasing, the activity of the immobilized lipase was decreased. This phenomenon may be attributed to the presence of spatial structural barriers preventing the enzyme from being activated when the glutaraldehyde concentration increased, because the modified PAN bound too much active aldehyde, and the enzyme molecules formed a multi-point binding with the carrier. The spatial organization of the enzyme’s active center may change due to an increase in the quantity of the enzyme bound to the active aldehyde, which would result in a reduction in enzyme activity [42]. Polyacrylonitrile treated with different methods was used as a support for some enzymes. In previous work, polyacrylonitrile was treated with hydrazine hydrochloride and activated with cyanuric chloride, and the immobilization efficiency was 81% [32]. Hydroxylamine hydrochloride was used to create an amidoxime group on the surface of polyacrylonitrile and utilized as a support for α-amylase with an immobilization efficiency of 79% [33].

### 2.2. Surface Characterization

**Figure 2:**
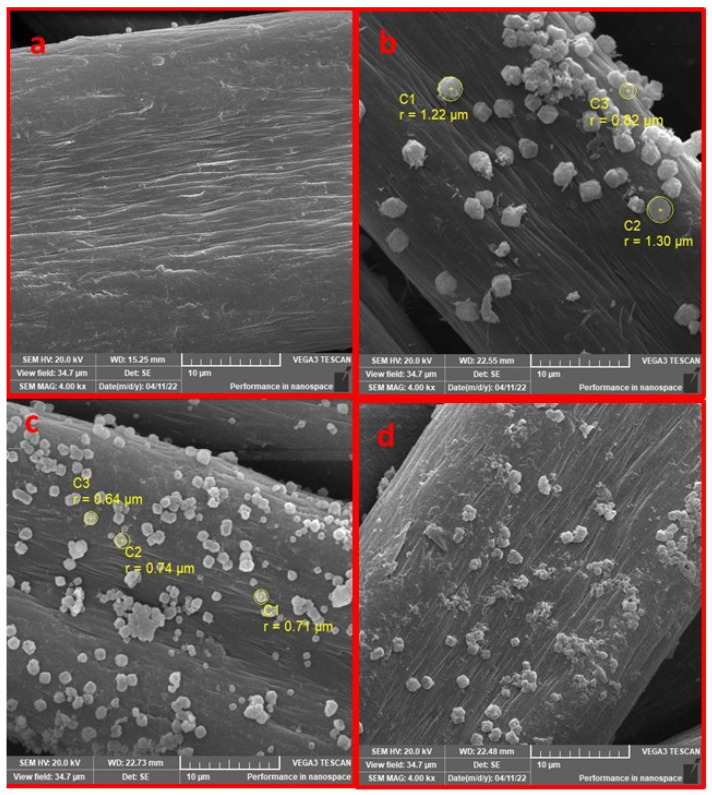
FESEM images of (**a**) pristine PAN, (**b**) PAN-HMDA, (**c**) PAN-HMDA-GL, and (**d**) PAN-HMDA-GL@Lipase.

The morphological properties of the pristine PAN, PAN-HMDA, PAN-HMDA-GL and PAN-HMDA-GL@Lipase enzyme were determined by FESEM analysis, presented in Figure 2a–d. In comparison to modified PAN, it was observed that the surface of pristine PAN was clear, smooth, and homogenous (Figure 2a). The SEM images revealed that a layer of HMDA, in small particles, was constructed on the surface of PAN, and the surface became rough, with randomly arranged particles. The small particles had a diameter of 0.82–1.30 μm (Figure 2b). After covalently crosslinking with glutaraldehyde, the particles became more regular, with agglomerations. The particles had a diameter of 0.64–0.71 μm (Figure 2c). A change in the surface of modified PAN was observed after enzyme immobilization. The heterogeneity and roughness of the surface increased, and it was clearly seen that the enzyme was successfully immobilized on the modified PAN surface (Figure 2d).

### 2.3. ATR-FTIR

**Figure 3:**
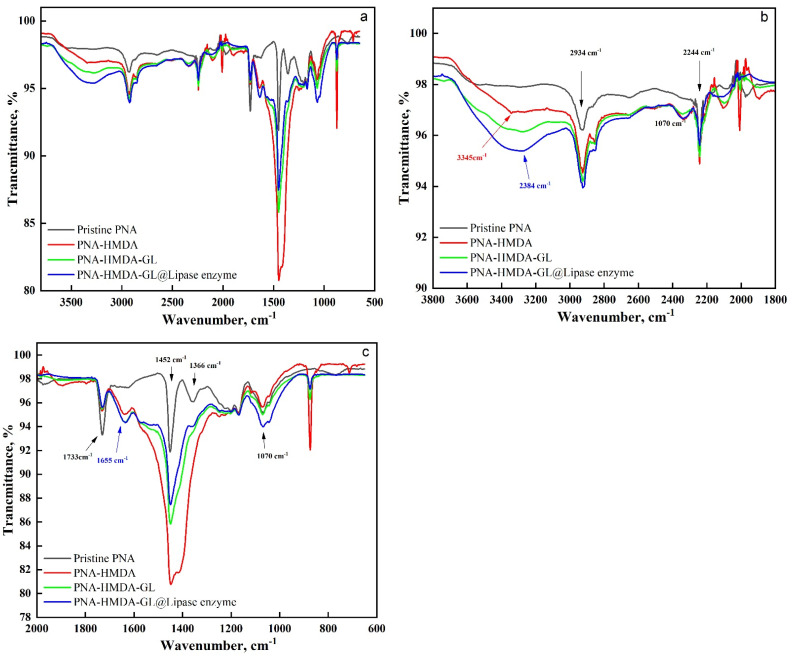
ATR-FTIR spectra of pristine PAN, PAN-HMDA, PAN-HMDA-GL, and PAN-HMDA-GL@Lipase ((**a**)-full scale, (**b**,**c**)-expanded scale).

Figure 3 shows the ATR-FTIR spectra of the enzyme-immobilized, HMDA-TA, glutaraldehyde-cross-linked, and pure acrylic samples. The characteristic bands of the acrylic sample appeared at 2934, 2244, 1733, 1070, 1452, and 1366 cm^−1^ due to CH_2_, CN, C=O, C-O-C, and C-H vibrations, respectively [43]. With the consecutive treatments, these bands were altered and changed in intensity. Interestingly, the HMDA-TA sample revealed a new broadband at 3345 cm^−1^ due to the NH_2_ and/or NH groups. Additionally, this sample displayed new bands at 1655 and a shoulder at 1557 cm^−1^ due to C=N with C=O amide III and NH, respectively. Furthermore, the HMDA-TA sample showed a strong and broad band centered around 1450 cm^−1^ due to the overlapped bands of groups, including the CH_2_, C-O, amide I, amide II, and NH groups. Interestingly, this strong band, upon glutaraldehyde crosslinking, appeared with lower intensity and little broadness. Furthermore, the intensity of the bands corresponding to the C=N and CH_2_ groups increased after crosslinking, indicating the success of the cross-linking. Upon enzyme immobilization, a large and broad band appeared near 3284 cm^−1^, due to the overlapped bands of the NH_2_ and OH groups present in the enzyme and the substrate. In addition, the C=N band appeared with high intensity owing to the formation of imine bonds between the enzyme and the substrate. Additionally, a new broadband was shown at 1072 cm^−1^ due to the C-O stretching vibration. Overall, the data confirmed the success of substrate activation and enzyme immobilization.

### 2.4. Reusability and Storage Stability

**Figure 4:**
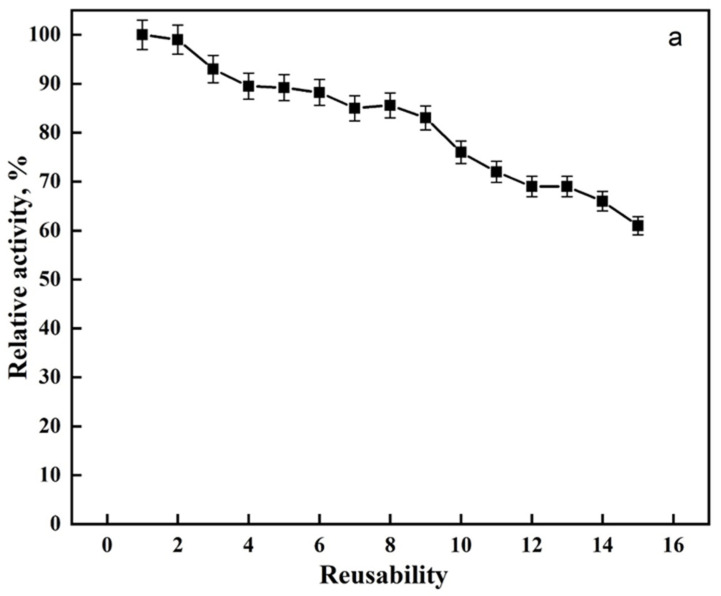

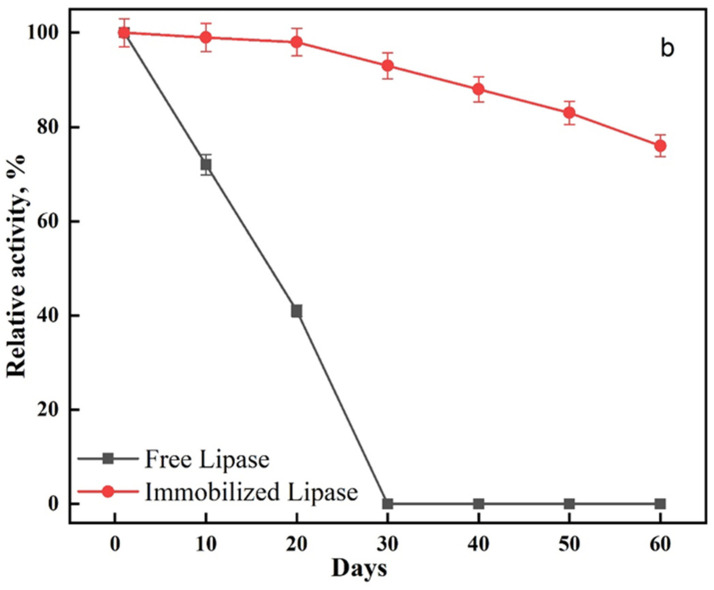
(**a**) Reusability and (**b**) storage stability of the lipase enzyme.

From a commercial standpoint, the reusability of an immobilized enzyme is a technologically important feature of applicable biocatalysts [44]. By monitoring the enzyme activity repeatedly, the reusability of immobilized lipase was investigated (15 times). Figure 4a shows that modified PAN allowed the maintenance of 76% of lipase activity after 10 reuses and 61% after 15 reuses. The improvement of reusability may be due to a covalent crosslinking between the enzyme and the modified PAN, avoiding denaturation or leakage of the enzyme. Our reusability findings were better compared to previous literature (Table 2).

The effect of storage stability on the free and immobilized lipase at 4 °C is shown in Figure 4b. According to the results, the immobilized lipase exhibited an extended storage stability compared to the free lipase. After 60 days, the immobilized lipase had 76% relative activity. In contrast, the free lipase lost its activity after 30 days. The improvement in stability may be due to the enzyme structure modification, which causes structural stiffness due to crosslinking [49]. Moreover, the enzyme becomes more resistant to agents that cause denaturation when bound to a matrix. As a result, an immobilized enzyme is more stable, which is related to a reduction in conformational flexibility.

### 2.5. Temperature and pH Properties

**Figure 5:**
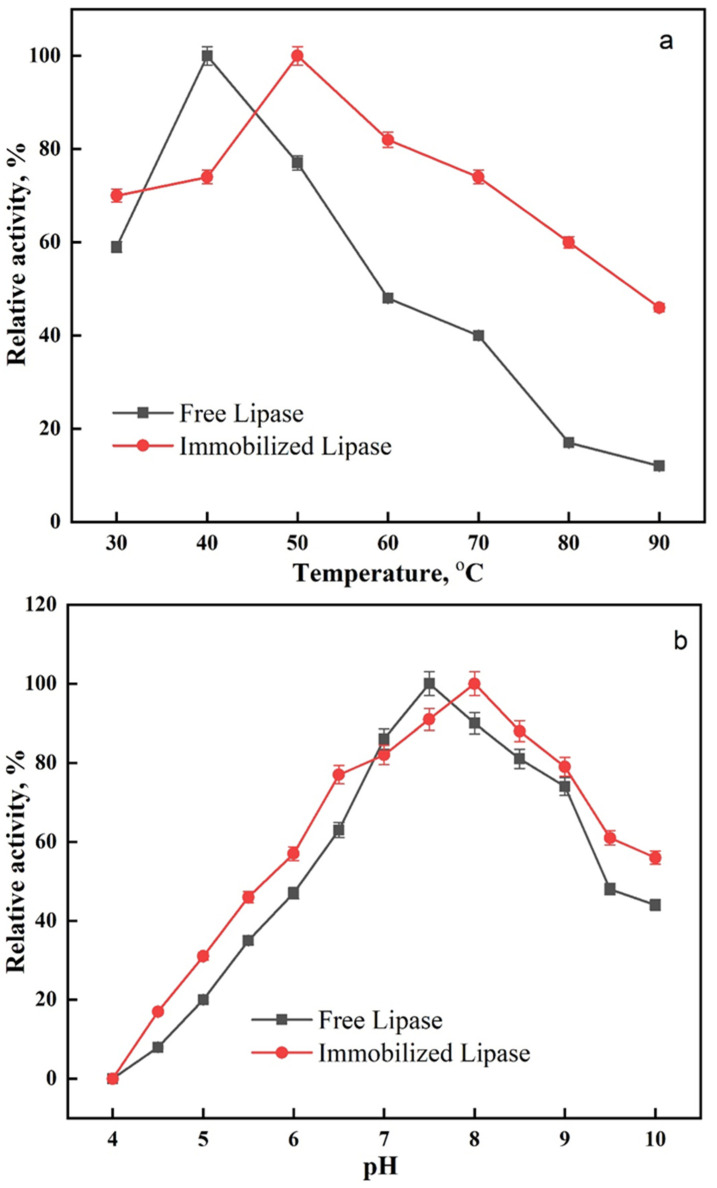
Effect of (**a**) temperature and (**b**) pH on the activity of free and immobilized lipase.

At different temperatures (30–90 °C), the activity profiles of free and immobilized lipase were investigated. It was shown that for the free and the immobilized lipase, the optimal temperatures were 40 °C and 50 °C, respectively (Figure 5a). Shifts in the optimal temperature values after immobilization have been reported in the literature. Lipase was immobilized on poly(vinyl alcohol)/Zn^2+^ by Işik et al. [50], who reported that the optimum temperature for both free and immobilized lipase fell within the wide range of 25–35 °C and 40–45 °C. Glutaraldehyde-activated poly(vinyl alcohol-co-ethylene) nanofibers were created by Zhu and Sun [51] to serve as lipase carriers, and the authors observed that the optimum temperatures for both free and immobilized lipases were 40 and 50 °C.

Varying the pH in the range from 4 to 10, the influence of pH on the activity of free and immobilized lipase was evaluated. The free and immobilized lipase exhibited their maximal activity at pH 7.5 and pH 8, respectively (Figure 5b). The shift in the optimum pH for the immobilized lipase agrees with recent studies [49,52] that showed comparable results after the formation of cross-linked enzyme aggregates.

### 2.6. Kinetic Parameters (Vmax and Km)

**Figure 6:**
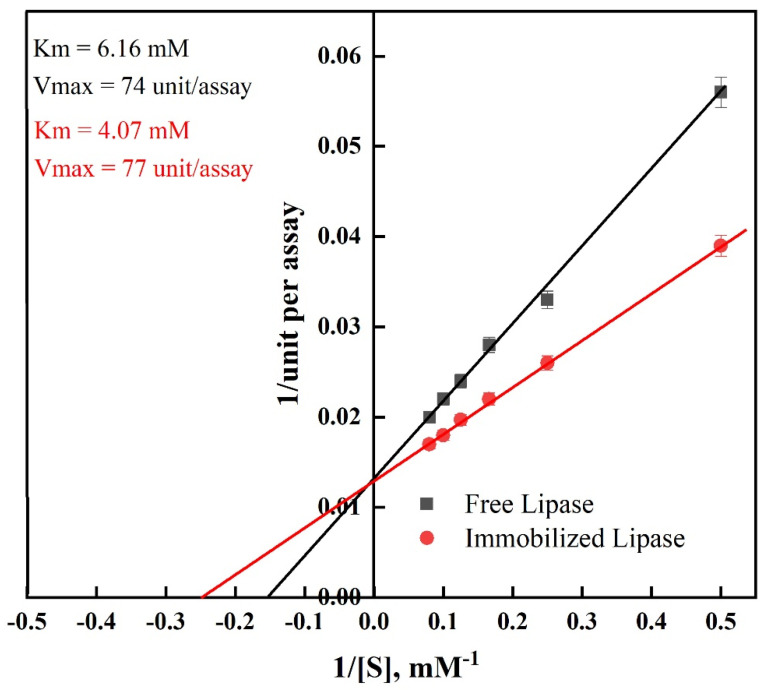
Kinetic parameters of free and immobilized lipase.

Lineweaver–Burk plots were utilized to determine the kinetic parameters for free and immobilized lipase. The affinity between the substrate and the enzyme is related to the Km. The apparent Km value of the immobilized lipase (4.07 mM), as shown in Figure 6, was lower than that of the free lipase (6.16 mM). This outcome was the consequence of the expansion of the enzyme on the surface of the modified PAN in a favorable location, which resulted in more reachable active sites and increased affinity to the lipase substrate [49]. The immobilized lipase had a greater Vmax than the free lipase. Consequently, the improvement in Vmax following the creation of the cross-linked enzyme aggregates indicated that less substrate was converted into a product per unit of time [53]. This could result from alterations in the enzyme structure following the covalent bonding with the cross-linker, which improved the stability of PAN-HMDA-GL@Lipase. 

## 3. Materials and Methods 

Lipase from *Candida rugosa*, *p*-nitrophenyl palmitate, and *p*-nitrophenol were purchased from Sigma-Aldrich (St. Louis, MI, USA). Hexamethylenediamine, 1,4- dioxane, sodium carbonate, and glutaraldehyde were provided by Merck (Darmstadt, Germany). Polyacrylonitrile was supplied by Misr El-Mahalla Co., El-Mahalla El-Kubra, Egypt, and contained 1/1 woven acrylic (40.6 × 40.6 threads inch^−1^ for both weft and warp), with a density of 0.36 g cm^−3^.

### 3.1. Polyacrylonitrile Treatment

The pre-treated sample was given a gentle water wash before being air-dried. One gram of PAN was soaked in 40 mL of dioxane solvent while being gently stirred, then 60 mL of HMDA 60% was added, followed by 0.5 g of sodium carbonate. The reaction mixture was heated to 110 °C and refluxed for 4 h in a paraffin oil bath. The treated sample was cleaned three times with distilled water and then treated with acetone before being allowed to dry by air. The treated sample (PAN-HMDA) was introduced to Falcon tubes containing 10 mL of glutaraldehyde at different concentrations (1–2.5%) prepared in 0.1 M phosphate-buffered saline and stirred for 4 h at room temperature. The modified samples (PAN-HMDA-GL) were washed with distilled water before air drying.

### 3.2. Lipase Immobilization

The obtained samples (PAN-HMDA-GL) were homogenized in 5 mg of lipase enzyme (80 units) in 50 mM sodium acetate buffer at pH 6.0 or Tris-HCl buffer at pH 7.0 or 8.0. The immobilization procedure took place for 12 h at room temperature. After collection, the samples (PAN-HMDA-GL@lipase) were washed with the same buffer and left to air-dry at room temperature. The Bradford technique was used to calculate the protein content, and bovine serum albumin was used as a reference [54]. According to the following equations, the immobilization yield and recovered activity were calculated:

$$\mathrm{Immobilization}\;\mathrm{Yield}\;(\mathrm{IY}\%)=\;\frac{\;\mathrm{Amount}\;\mathrm{of}\;\mathrm{protein}\;\mathrm{introduced}\;-\;\mathrm{Protein}\;\mathrm{in}\;\mathrm{the}\;\mathrm{supernatant}}{\mathrm{Amount}\;\mathrm{of}\;\mathrm{protein}\;\mathrm{introduced}\;}\times 100$$

$$\mathrm{Activity}\;\mathrm{yield}\;(\mathrm{AY}\%)=\frac{\mathrm{Immobilized}\;\mathrm{enzyme}\;\mathrm{activity}}{\mathrm{Iniatial}\;\mathrm{activity}}\times 100$$

### 3.3. Lipase Activity Assay

The activity of free and immobilized lipase was determined by determining the quantity of *p*-nitrophenol (*p*-NP) produced from the reaction mixture (1 mL) containing 5 mM *p*-nitrophenyl palmitate (*p*-NPP) dissolved in isopropanol, 50 mM Tris-HCl buffer (pH 8.0), and 20 mg of immobilized enzyme or the least amount of free enzyme. The reaction mixture was incubated for 10 min at 50 °C. Finally, to stop the reaction, 500 µL of sodium carbonate (2 mM) was added. The yellow color produced from the release of *p*-NP in the solution was recorded at 410 nm. One unit of lipase activity is defined as the amount of enzyme producing 1 μmol *p*-nitrophenol per min. 

### 3.4. Characterization of the Solid Support

The surface morphology of the solid support before and after immobilization was analyzed using field-emission scanning electron microscopy (FESEM) (Quanta FEG 250, FEI Co., Hillsboro, OR, USA). The chemical composition of the modified polyacrylonitrile was analyzed using Fourier-transform infrared spectroscopy (FTIR, PerkinElmer Spectrum 100).

### 3.5. Influence of Temperature and pH

The influence of the temperature on free and immobilized lipase activity was evaluated at temperatures ranging from 30 to 80 °C. The influence of pH on the activity of free and immobilized lipase was determined using 50 mM sodium acetate (pH 4–6.0), Tris-HCl (pH 6.5–9), and carbonate/bicarbonate (pH 9.5–10). The relative activity was determined according to the following equation:

Relative activity, (%) = (OD_x_/OD_a_) ∗ 100

where ODx is the lower absorbance, and ODa is the higher absorbance.

### 3.6. Reusability and Storage Stability

Enzyme reusability in industrial applications is an important requirement for effective operations. After each cycle, the immobilized enzyme was withdrawn from the reaction mixture using tweezers and washed with Tris-HCl buffer (50 mM, pH 8.0). The relative lipase activity (%) was measured as the ratio of the residual lipase activity to the initial lipase activity. The storage stability of free and immobilized lipase was measured at 4 °C for 60 days, and the relative activity was measured.

### 3.7. Kinetics Analysis

The initial reaction rates with *p*-NPP were used to calculate the kinetic parameters of free and immobilized lipase. The Michaelis–Menten equation was used to determine the values of Km and Vmax.

## 4. Conclusions 

To the best of our knowledge, for the first time, PAN modified by HMDA was effectively utilized to immobilize lipase. It was observed that covalent crosslinking between modified PAN and lipase considerably improved the catalytic performance, reusability, storage stability, and enzymatic activity of lipase on modified PAN. The immobilization yield of the lipase was 81%, and the activity was 91% at pH 8.0. By studying the enzymatic reaction kinetics (Vmax and Km) based on the Michaelis–Menten model, it was possible to determine the affinity of the substrate for the enzyme and some of its inherent features. A reduced Km value suggested that modified PAN improved the lipase’s affinity for the substrate and its immobilization. The results demonstrated that synthetically modified PAN is appropriate for immobilizing enzymes and has a potential for commercial applications.

## Figures and Tables

**Table 1:** Activity yields (AY) and immobilization yields (IY) of lipase loaded on modified PAN.

	Treatment with Glutaraldehyde	Activity Yield, %	Immobilization Yield, %
pH 6	1%	72 ± 0.96	75 ± 0.89
	1.5%	59 ± 0.57	53 ± 0.83
	2%	58 ± 0.25	48 ± 0.64
	2.5%	53 ± 0.48	43 ± 0.49
pH 7	1%	81 ± 1.05	77 ± 0.53
	1.5%	65.5 ± 0.44	61 ± 0.68
	2%	68 ± 0.62	63 ± 0.49
	2.5%	54.5 ± 0.76	38 ± 0.58
pH 8	1%	91 ± 1.11	81 ± 0.99
	1.5%	58 ± 0.59	72 ± 0.45
	2%	55 ± 0.63	62 ± 0.82
	2.5%	49 ± 0.49	31 ± 0.67

**Table 2:** Comparison of the reusability of the lipase enzyme immobilized on different modified nanofibers.

Nanofibers	Number of Reuse (Residual Activity)	Immobilization Method	Reference
Polyacrylonitrile	8 (75%)	Covalent	[45]
Polyacrylonitrile	10 (50%)	Covalent	[46]
Polyacrylonitrile	10 (50%)	Encapsulation	[47]
Polyvinyl alcohol/alginate	14 (50%)	Adsorption + cross-linking	[48]
The current work	15 (61%)	covalently crosslinking	-

## Data Availability

Data are contained within the article.
